# Use of Health Care Services and Its Associated Factors Among People With Type 2 Diabetes in Nepal

**DOI:** 10.1177/10105395241236058

**Published:** 2024-03-10

**Authors:** Grish Paudel, Corneel Vandelanotte, Padam Kanta Dahal, Lal Rawal

**Affiliations:** 1 School of Health, Medical and Applied Sciences, Central Queensland University, Sydney, NSW, Australia; 2 Appleton Institute, Physical Activity Research Group, Central Queensland University, Rockhampton, QLD, Australia; 3 Translational Health Research Institute (THRI), Western Sydney University, Sydney, NSW, Australia

**Keywords:** Nepal, type 2 diabetes mellitus, utilization of health care services

## Abstract

This study aimed to assess the utilization of health care services and its associated factors among people with type 2 diabetes mellitus (T2DM) in Nepal. Data on the utilization of health care services were assessed in 481 adults aged 30 to 70 years with T2DM in Nepal. Multiple logistic regression analysis was performed to determine the factors associated with the utilization of health care services. Over 6 months, 66.1% of participants visited health care facilities or health service providers, followed by specialist visits (3.5%), hospitalization (2.1%), and emergency department visits (1.9%). Visit to health care facilities was significantly higher among those aged 50 to 59 years old (OR_A_: 1.64), practicing Hinduism (OR_A_: 2.4), and earning NRs ≥30 000 (≥USD 226.10) (OR_A_: 1.82) as compared to those aged ≥60 years old, practicing other religions, and with monthly family income NRs ≤10 000 (≤USD 75.37), respectively. The utilization of health care services among people with T2DM in Nepal was reasonably low. Identifying the underlying causes of low use of health care services is of great importance to bridge the gap in using health care services for management of diabetes.

## What We Already Know

- Type 2 diabetes mellitus (T2DM) has significantly increased across the world, mostly in low- and middle-income countries in recent decades.
- Existing evidence has shown that people with diabetes tend to visit physicians or health care providers, specialists, and emergency rooms more frequently than those without diabetes and are also more likely to be hospitalized.

## What This Article Adds

- Visit to health care facilities within the last 6 months was 66.1% among the people with T2DM living in rural and urban areas of Nepal. Visit to specialists, emergency departments, and hospital admission was generally low among the study participants.
- Identifying the underlying causes of low use of health care services is of great importance to bridge the gap in the use of health care services and the management of diabetes.

## Introduction

Diabetes mellitus is a chronic health condition that affects various organs leading to premature deaths and disability.^1^ In recent years, the prevalence of diabetes among the adult population (20-79 years) in Nepal has increased from 488 200 in 2011 to 1.1 million in 2021.^1^ The effective management of diabetes can delay or prevent life-threatening health complications such as cardiovascular diseases, neuropathy, nephropathy, and foot and eye-related diseases.^1^

The utilization of health care services plays a significant role in managing type 2 diabetes mellitus (T2DM) by enhancing the interaction between health care providers and patients, thereby improving diabetes care and control.^2^ However, the availability of diabetes-specific services through interprofessional collaboration between the service providers is not organized and uniform in Nepal.^3^ The majority of diabetes services in Nepal are delivered by the private sector and are urban-centered.^3^ Moreover, a systematic review from Nepal highlighted that the existing knowledge on the burden of diabetes, its treatment, and control is still insufficient in Nepal.^4^ Insufficient services and resources for diagnosis, treatment, and prevention of diabetes at the primary health care level are major causes of higher disease burden in Nepal.^3^

Understanding the health care service utilization among people with diabetes is an urgent priority to generate evidence-based recommendations to health authorities in countries like Nepal where diabetes services or resources are inadequate. Hence, the aim of this study was to assess the utilization of health care services and its associated factors among people with type 2 diabetes in Nepal.

## Methods

### Study Design and Participants

This study includes cross-sectional data from a randomized controlled trial (RCT) implemented in rural and urban communities of Nuwakot and Kavrepalanchowk districts of Nepal. The detailed methodology of the RCT has been published elsewhere.^5^ In brief, this study uses baseline data, collected between July 2021 and February 2022, from the RCT, which has 481 participants at baseline. Those aged 30 to 70 years who were clinically diagnosed with T2DM were enrolled in the study.

### Study Measures

#### Measure of dependent variable

The utilization of health care services among people with type 2 diabetes was assessed by asking the participants if they had visited (1) a physician or health facility, (2) an emergency department, (3) a specialist visit, and (4) if they were hospitalized due to the complications of type 2 diabetes during the last six months. The answers to all the primary outcome variables were categorized as “No” and “Yes” for the logistic regression analysis; however, all the continuous findings are reported in the descriptive findings in Table 1.

**Table 1.** General Characteristics of Study Participants and Health Care Service Use by Gender.

Characteristics	Frequency (%)	Gender	
		Male (n = 254)	Female (n = 227)
**General characteristics of the study participants**			
**Predisposing factors**			
**Area of residence**			
Rural	172 (35.8%)	92 (36.2%)	80 (35.2%)
Urban	309 (64.2%)	162 (63.8%)	147 (64.8%)
**Age (Years) [mean: 54.44, SD: ±9.42]**			
30-39	39 (8.1%)	19 (7.5%)	20 (8.8%)
40-49	94 (19.5%)	52 (20.5%)	42 (18.5%)
50-59	186 (38.7%)	101 (39.8%)	85 (37.4%)
≥60	162 (33.7%)	82 (32.3%)	80 (35.2%)
**Ethnicity**			
Brahmin/Chhetri	240 (49.9%)	131 (51.6%)	109 (48%)
Newar/Janajati	208 (43.2%)	105 (41.3%)	103 (45.4%)
Others (Dalit/Madhesi)	33 (6.9%)	18 (7.1%)	15 (6.6%)
**Religion**			
Hindu	429 (89.2%)	229 (90.2%)	200 (88.1%)
Others (Buddhist/Christian)	52 (10.8%)	25 (9.8%)	27 (11.9%)
**Marital status**			
Currently married	447 (92.9%)	247 (97.2%)	200 (88.1%)
Separated/Widowed/never married	34 (7.1%)	7 (2.8%)	27 (11.9%)
**Living arrangement**			
Alone	12 (2.5%)	3 (1.2%)	9 (4%)
With family members	469 (97.5%)	251 (98.8%)	218 (96%)
**Educational level**			
No formal education	199 (41.4%)	37 (14.6%)	162 (71.4%)
Below high school level (below grade 10)	138 (28.7%)	99 (39%)	39 (17.2%)
Secondary level and higher (grade 10 and above)	144 (29.9%)	118 (46.5%)	26 (11.5%)
**Current use of tobacco (Smoking and/or smokeless)**			
No	347 (72.1%)	157 (61.8%)	190 (83.7%)
Yes (Daily and occasionally)	134 (27.9%)	97 (38.2%)	37 (16.3%)
**Current use of alcohol**			
No	372 (77.3%)	177 (69.7%)	195 (85.9%)
Yes	109 (22.7%)	77 (30.3%)	32 (14.1%)
**Enabling factors**			
**Main occupation**			
Household chores	106 (22%)	0	106 (46.7%)
Agriculture/animal husbandry	206 (42.8%)	124 (48.8%)	82 (36.1%)
Business/service	112 (23.3%)	76 (29.9%)	36 (15.9%)
Others (wage/labor/driver/retired)	57 (11.9%)	54 (21.3%)	3 (1.3%)
**Monthly family income**			
≤10 000 NRs (≤$75.37)	125 (26%)	54 (21.3%)	71 (31.3%)
10 001-19 999 NRs ($75.38-$150.73)	73 (15.2%)	39 (15.4%)	34 (15%)
20 000-29 999 NRs ($150.74-$226.10)	91 (18.9%)	48 (18.9%)	43 (18.9%)
≥30 000 NRs (≥$226.10)	192 (39.9%)	113 (44.5%)	79 (34.8%)
**Health insurance**			
No	267 (55.5%)	136 (53.5%)	131 (57.7%)
Yes	214 (44.5%)	118 (46.5%)	96 (42.3%)
**Duration of T2DM [5.3 ±5.4]**			
≤5 years	302 (62.8%)	160 (63%)	142 (62.6%)
>5 years	179 (37.2%)	94 (37%)	85 (37.4%)
**Availability of free health services**			
No	378 (78.6%)	205 (80.7%)	173 (76.2%)
Yes	103 (21.4%)	49 (19.3%)	54 (23.8%)
**Distance to health facility**			
≤30 minutes	310 (64.4%)	159 (62.6%)	151 (66.5%)
>30 minutes	171 (35.6%)	95 (37.4%)	76 (33.5%)
**Waiting time to make a new appointment**			
Less than a day	414 (86.1%)	205 (80.7%)	209 (92.1%)
One day	54 (11.2%)	40 (15.7%)	14 (6.2%)
2-7 days	13 (2.7%)	9 (3.5%)	4 (1.8%)
**Waiting time at the health facility**			
≤30 minutes	218 (45.3%)	120 (47.2%)	98 (43.2%)
>30 minutes	263 (54.7%)	134 (52.8%)	129 (56.8%)
**Ever been to traditional healers for treating DM**			
No	462 (96%)	246 (96.9%)	216 (95.2%)
Yes	19 (4%)	8 (3.1%)	11 (4.8%)
**Need-related factors**			
**Comorbid conditions** ^#^			
No	243 (50.5%)	131 (51.6%)	112 (49.3%)
Yes	238 (49.5%)	123 (48.4%)	115 (50.7%)
**Self-rated health status**			
Good	226 (47%)	134 (52.8%)	92 (40.5%)
Fair/satisfactory	228 (47.4%)	111 (43.7%)	117 (51.5%)
Poor	27 (5.6%)	9 (3.5%)	18 (7.9%)
**Body mass index (BMI)**			
Underweight (<18.5 kg/m^2^)	7 (1.5%)	4 (1.6%)	3 (1.3%)
Normal (18.5-22.9 kg/m^2^)	73 (15.2%)	46 (18.1%)	27 (11.9%)
Overweight (23-24.9 kg/m^2^)	98 (20.4%)	57 (22.4%)	41 (18.1%)
Obese (≥25 kg/m^2^)	303 (63%)	147 (57.9%)	156 (68.7%)
**Glycated hemoglobin (HbA1c level)**			
Uncontrolled (HbA1c ≥7%)	321 (66.7%)	164 (64.6%)	157 (69.2%)
Controlled (HbA1c <7%)	160 (33.3%)	90 (35.4%)	70 (30.8%)
**Diabetes-related health care service utilization during the last 6 months**			
**Number of visits to health service provider or physician**			
Zero	163 (33.9%)	84 (33.1%)	79 (34.8%)
One	132 (27.4%)	64 (25.2%)	68 (30%)
Two	75 (15.6%)	41 (16.1%)	34 (15%)
Three	56 (11.6%)	31 (12.2%)	25 (11%)
Four	23 (4.8%)	15 (5.9%)	8 (3.5%)
Five or more	32 (6.7%)	19 (7.5%)	13 (5.7%)
**Number of emergency department (ED) visits**			
Zero	472 (98.1%)	248 (97.6%)	224 (98.7%)
One	7 (1.5%)	4 (1.6%)	3 (1.3%)
Two	2 (0.4%)	2 (0.8%)	0
**Number of overnight hospital stays**			
Zero	471 (97.9%)	246 (96.9%)	225 (99.1%)
One	7 (1.5%)	5 (1.9%)	2 (0.9%)
Two	2 (0.4%)	2 (0.8%)	0
Three	1 (0.2%)	1 (0.4%)	0
**Number of days admitted (n = 10)**			
Up to 4 days	4 (40%)	4 (50%)	0
≥5 days	6 (60%)	4 (50%)	2 (100%)
**Number of specialist visits**			
Zero	464 (96.5%)	245 (96.5%)	219 (96.5%)
One	14 (2.9%)	7 (2.7%)	7 (3.1%)
Two	3 (0.6%)	2 (0.8%)	1 (0.4%)

NRs: Nepali Rupee.

$: United States Dollar.

Exchange rate: 1 USD = NRs 132.68 as of January 15, 2024, exchange rate.

# Comorbid conditions include any of the following health conditions: heart problems, stroke, asthma, lung diseases, kidney problems, depression/anxiety, back problems, arthritis, osteoporosis, cancer, high blood cholesterol, and high blood pressure.

#### Measure of independent variable

Several independent variables were identified through a review of the literature and were categorized into predisposing, enabling, and need of care factors adapting the Andersen Behavioral Framework for Health Service Utilization.^6^ In the model, predisposing factors refer to the sociodemographic characteristics such as age, gender, ethnicity, religion, marital status, education, and living arrangements; enabling factors refer to services or resources that facilitate the use of the services such as occupation, income, health insurance, availability of free health care services, distance to the health facility, and waiting hours; and need factors refer to physical condition and disease characteristics that motivate service use, which includes comorbid conditions, self-rated health status, body mass index (BMI), and glycated hemoglobin (HbA1c).

### Statistical Analysis

Data analysis was performed using the IBM SPSS version 23. Multivariate logistic regression was performed to identify the variables associated with visits to health care facilities or service providers during the last 6 months. Visits to specialists, emergency departments, and hospitalization were very low, and therefore not analyzed using logistic regression, as it may lead to biased estimates and inaccurate inferences.

## Results

**Figure 1.**
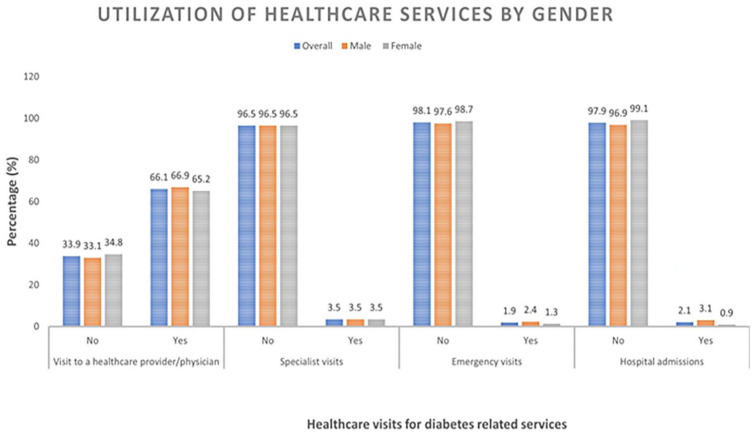
Utilization of health care services to manage type 2 diabetes.

The mean (±SD) age of the study participants was 54.4 (± 9.4) years and 52.8% were males (Table 1). The majority of the study participants (66.1%) visited a health care facility or service provider within the last 6 months preceding the survey (Figure 1). Age, religion, occupation, and monthly family income were significantly associated with visits to health care facility or service provider within the last 6 months (Table 2).

**Table 2.** Multivariate Regression Analysis to Determine Predictors of the Visit to Health Facility or Service Provider Within the Last 6 Months.

Characteristics	Visit to health facility or service provider within the last 6 months	
	Adjusted odds ratio	*P*-value
**Predisposing factors**		
**Area of residence**		
Rural	Ref	
Urban	1.32 (0.84 - 2.08)	.233
**Gender**		
Male	1.3 (0.7 - 2.43)	.412
Female	Ref	
**Age**		
30-39 years	2.09 (0.83 - 5.26)	.116
40-49 years	0.86 (0.47 - 1.56)	.61
50-59 years	1.64 (1.01 - 2.67)	**<.05**
60 and above	Ref	
**Ethnicity**		
Brahmin/Chhetri	0.64 (0.26 - 1.58)	.336
Newar/Janajati	0.89 (0.37 - 2.13)	.789
Dalit/Madhesi	Ref	
**Religion**		
Hindu	2.4 (1.2 - 4.81)	**<.05**
Others (Buddhist/Christian)	Ref	
**Marital status**		
Currently married	1.26 (0.55 - 2.9)	.587
Separated/widowed/never married	Ref	
**Living arrangement**		
Alone	Ref	
Family	1.1 (0.27 - 4.55)	.892
**Educational level**		
No formal education	Ref	
Under secondary level (under grade 10)	1.24 (0.68 - 2.27)	.483
Secondary level and higher (grade 10 and above)	0.95 (0.49 - 1.85)	.884
**Tobacco use**		
No	Ref	
Yes	1.41 (0.86 - 2.3)	.168
**Alcohol use**		
No	Ref	
Yes	0.81 (0.48 - 1.37)	.438
**Enabling factors**		
**Main occupation**		
Household chores	Ref	
Agriculture/animal husbandry	0.76 (0.41 - 1.41)	.383
Business/service	0.4 (0.2 - 0.82)	**<.05**
Others (wages/labor/retired)	0.41 (0.17 - 0.98)	**<.05**
**Monthly family income**		
≤10 000 NRs (≤$76.17)	Ref	
10 001-19 999 NRs ($76.18-$152.34)	1.59 (0.83 - 3.06)	.166
20 000-29 999 NRs ($152.34-$228.51)	1.19 (0.64 - 2.21)	.589
≥30 000 NRs (≥$228.52)	1.82 (1.05 - 3.15)	**<.05**
**Health insurance**		
No	Ref	
Yes	1.16 (0.72 - 1.89)	.543
**Availability of free health services**		
No	Ref	
Yes	1.09 (0.63 - 1.86)	.765
**Duration of T2DM**		
≤5 years	Ref	
>5 years	0.88 (0.57 - 1.36)	.565
**Distance to health facility**		
≤30 minutes	0.81 (0.51 - 1.28)	.36
>30 minutes	Ref	
**Waiting time to make a new appointment**		
Less than a day	1.21 (0.67 - 2.21)	.524
One day or more	Ref	
**Waiting time at health facility**		
≤30 minutes	1.02 (0.65 - 1.59)	.934
>30 minutes	Ref	
**Need factors**		
**Comorbid conditions (other co-existing health conditions)**		
No	Ref	
Yes	0.73 (0.48 - 1.12)	.146
**Self-rated health status**		
Good	Ref	
Fair/satisfactory	1.05 (0.69 - 1.61)	.814
Poor	1.33 (0.52 - 3.39)	.554
**Body mass index**		
Normal	Ref	
Underweight	0.19 (0.04 - 1.08)	.061
Overweight/obese	0.88 (0.49 - 1.58)	.664
**Glycated hemoglobin level**		
Uncontrolled (HbA1c ≥7%)	Ref	
Controlled (HbA1c <7%)	1.37 (0.8 - 2.35)	.244

The prevalence of visits to the emergency department (1.9%), visits to specialists (3.5%), and hospital stays (2.1%) was very low.

## Discussion

In this study, 66% of participants visited health care facilities, which is much lower than a previously conducted study in Nepal (93.7%).^7^ The findings of this study show that the utilization of health care services among people with T2DM in Nepal is reasonably low, which requires immediate attention to prevent the progression of early diabetic complications such as kidney disease, artery diseases, neuropathy, eye problems, and foot ulceration.

Similarly, despite a low level of health care service use in our study, a lower proportion of participants visited the emergency department and were hospitalized compared with previously conducted study in Nepal.^7^ This might be because people in Nepal tend to seek medical services unless they are severely sick and some often terminate the treatment of their chronic condition due to unaffordability of the health care services (high cost of medication and inaccessible health care services).^8^

In this study, age, religion, family income, and occupation are the only factors associated with visiting a health care facility, and this is comparable to findings of other studies.^9,10^ However, none of the need factors were associated with the health care use in our study. These discrepancies might be because higher proportion (94.4%) of study participants in our study self-rated their health as good or satisfactory and more than half (50.5%) were without comorbid conditions which might have resulted in a lower visit to the health facility.

This study is limited by its reliance on self-reported survey data, which may lead to recall bias and a causal relationship between the variables could not be established due to the cross-sectional nature of the survey. Despite these limitations, this is the first community-based study to assess the utilization of health care services and its associated factors among people with T2DM from rural and urban population of Nepal. We conclude that the utilization of health care services among people with T2DM in Nepal was reasonably low. Identifying the underlying causes of low use of health care services is of great importance to bridge the gap in health care service use and management of diabetes.
